# Combined detection of CXCR7 and CXCL12 for diagnosing acute myocardial infarction and predicting post-percutaneous coronary intervention major adverse cardiovascular events

**DOI:** 10.5937/jomb0-61266

**Published:** 2026-04-15

**Authors:** Hong Pan, Xiaoli Fan, Yu Liu, Gulijiakela Aishan, Jingwen Shao

**Affiliations:** 1 Fudan University, Huashan Hospital, Department of Emergency, Shanghai, China; 2 Gongli Hospital of Shanghai Pudong New Area, Department of Rehabilitation Medicine, Shanghai, China

**Keywords:** CXCR7, CXCL12, acute myocardial infarction, major adverse cardiovascular events, combined diagnosis, CXCR7, CXCL12, akutni infarkt miokarda, veliki neželjeni kardiovaskularni događaji, kombinovana dijagnoza

## Abstract

**Background:**

To explore the diagnostic utility of combined CXCR7 and CXCL12 testing in acute myocardial infarction (AMI) and its predictive value for post-percutaneous coronary intervention (PCI) major adverse cardiovascular events (MACE).

**Methods:**

This study enrolled 164 AMI patients and 54 healthy controls from January to June 2024. All patients received standardized PCI and were followed for 12 months. Serum CXCR7 and CXCL12 concentrations were determined via enzyme-linked immunosorbent assay (ELISA), while NT-proBNP was quantified with an automated biochemical analyzer. Comparative analyses of biomarker levels between groups were conducted, along with receiver operating characteristic (ROC) curve analysis for combined diagnostic performance. Dynamic changes in CXCR7/CXCL12 were also assessed in relation to postoperative MACE.

**Results:**

Pre-treatment concentrations of CXCR7 and CXCL12 were markedly higher in the AMI group relative to controls. The combined detection achieved an AUC value of 0.812 for diagnosing AMI, superior to individual indicators (P&lt; 0.05). Both markers decreased significantly following PCI (P&lt; 0.05). Those who developed MACE exhibited higher post-treatment CXCR7 and CXCL12 levels compared to non-MACE cases; their combined AUC reached 0.806 for predicting post-PCI MACE (P&lt; 0.05). While NT-proBNP demonstrated strong diagnostic accuracy for AMI (AUC = 0.893), it failed to predict post-PCI MACE (P&gt; 0.05). This discrepancy may reflect NT-proBNP's primary association with acute myocardial injury rather than chronic microvascular dysfunction or endothelial repair processes implicated in MACE.

**Conclusions:**

The favorable diagnostic performance of CXCR7 and CXCL12 for AMI and post-PCI MACE suggests their potential as a novel protocol for the clinical assessment of AMI.

## Abstract (sr)

**Uvod:**

Cilj je istražiti dijagnostičku korisnost kombinovanog testiranja CXCR7 i CXCL12 kod akutnog infarkta miokarda (AIM) i njegovu prediktivnu vrednost za glavne neželjene kardiovaskularne događaje (MACE) nakon perkutane koronarne intervencije (PCI).

**Metode:**

Ova studija je obuhvatila 164 pacijenta sa amitorejtivnim infarktom miokarda i 54 zdrave kontrolne osobe od januara do juna 2024. godine. Svi pacijenti su primili standardizovanu perkutanu perkutanu intervenciju (PCI) i praceni su 12 meseci. Koncentracije serumskih CXCR7 i CXCL12 određene su enzimski povezanim imunosorbentnim testom (ELISA), dok je NT-proBNP kvantifikovan automatizovanim biohemijskim analizatorom. Sprovedene su uporedne analize nivoa biomarkera između grupa, zajedno sa analizom ROC krive za kombinovane dijagnostičke performanse. Dinamičke promene CXCR7/CXCL12 su takođe procenjene u odnosu na postoperativni MACE.

**Rezultati:**

Koncentracije CXCR7 i CXCL12 pre tretmana bile su znatno veće u grupi sa AIM u odnosu na kontrolnu grupu. Kombinovana detekcija postigla je vrednost AUC od 0,812 za dijagnostikovanje AIM, što je superiornije u odnosu na pojedinačne indikatore (P&lt;0,05). Oba markera su se značajno smanjila nakon PCI (P&lt;0,05). Oni koji su razvili MACE pokazali su veće nivoe CXCR7 i CXCL12 nakon tretmana u poređenju sa slučajevima koji nisu imali MACE; njihova kombinovana AUC je dostigla 0,806 za predviđanje MACE nakon PCI (P&lt;0,05). Dok je NT-proBNP pokazao snažnu dijagnostičku tačnost za AIM (AUC=0,893), nije uspeo da predvidi MACE nakon PCI (P&gt;0,05). Ova razlika može odražavati primarnu povezanost NT-proBNP sa akutnim oštećenjem miokarda, a ne sa hroničnom mikrovaskularnom disfunkcijom ili procesima endotelne popravke impliciranim u MACE.

**Zaključak:**

Povoljne dijagnostičke performanse CXCR7 i CXCL12 za amitorejtivni infarkt miokarda (AIM) i značajne egzistencijalne intervencije (MACE) nakon perkutanne perkutane koronarne intervencije (PCI) ukazuju na njihov potencijal kao novog protokola za kliničku procenu AIM.

## Introduction

Acute myocardial infarction (AMI) stands as the leading cardiovascular emergency causing mortality and disability worldwide [1]. In China alone, the annual incidence of AMI has surpassed 1.5 million, with a noticeable trend toward affecting younger age groups [2]. Percutaneous coronary intervention (PCI), the preferred reperfusion strategy for AMI, rapidly opens infarct-related arteries and salvages endangered myocardium. However, about 15-20% of patients still suffer from major adverse cardiovascular events (MACE) within 3-12 months post-operation [3]. Existing clinical tools for MACE risk assessment rely heavily on subjective scores and imaging-based markers. For example, the Global Registry of Acute Coronary Events (GRACE) relies heavily on clinical variables (age, troponin), and the Global Registry of Acute Coronary Events (GRACE) relies heavily on clinical variables (age, troponin). Lack of dynamic evaluation of coronary microenvironment repair [4]. The low predictive accuracy of GRACE for MACE [5] highlights the need for biomarkers that reflect the pathophysiological evolution after PCI. Thus, creating precise risk prediction models for post-PCI MACE to enable early identification and individualized intervention is an urgent priority in cardiovascular medicine.

As precision medicine advances, biomarkers have gained increasing value in risk assessment. High-sensitivity troponin and N-terminal pro-B-type natriuretic peptide (NT-proBNP), despite correlating with myocardial injury severity, show restricted specificity for prognosticating long-term post-PCI outcomes [6]. Similarly, inflammatory factors, though reflecting systemic inflammation, fail to specifically indicate abnormal repair of the coronary microenvironment [7]. CXC chemokine receptor 7 (CXCR7), recognized as a »non-classical« chemokine receptor, has gained increasing research interest in recent years, with CXCL12 being its principal ligand [8]. The CXCR7/CXCL12 axis orchestrates myocardial repair through dual mechanisms: (1) pro-angiogenic signaling: CXCL12 binding to CXCR7 activates the PI3K/Akt pathway, enhancing EPC proliferation and homing to ischemic myocardium [9]; (2) anti-inflammatory effects: CXCR7 ligation suppresses NF-kB activation, reducing neutrophil infiltration and IL-6/TNF-α production [9]. Importantly, animal research shows that post-AMI CXCR7 expression correlates positively with myocardial repair efficiency, while CXCR7-knockout mice exhibit increased susceptibility to ventricular remodeling [10]. These findings support the potential of CXCR7 and CXCL12 as MACe assessment indicators, though their exact clinical utility warrants further investigation.

This study investigates the prognostic significance of CXCR7 and CXCL12 for post-PCI MACE in AMI patients. This exploration aims to improve early risk stratification, assist clinicians in providing targeted therapy for high-risk individuals, and advance precision medicine in cardiovascular care.

## Materials and methods

### Study participants

This study enrolled AMI patients and healthy controls who received physical examinations at our hospital between January and June 2024. Sample size estimation, derived from preliminary CXCR7 data (median: 2.1 ng/mL in AMI vs. 1.2 ng/mL in controls, SD = 0.8) and assuming 80% diagnostic sensitivity and 75% specificity, indicated that a minimum of 216 participants were required to achieve an area under the curve (AUC)>0.85. α = 0.05 (type I error rate), β = 0.2 (type II error rate, corresponding to 80% power). The final cohort comprised 164 AMI patients and 54 healthy controls.

Inclusion criteria: ① Meeting AMI diagnostic criteria [11], with examination confirmation; ② Successfully receiving PCI during hospitalization [Successful PCI was defined as achievement of thrombolysis in myocardial infarction (TIMI) flow grade 2b post-procedure, with residual stenosis <30% on angiography]; ③ Aged 18-80 years, providing informed consent, and agreeing to baseline data collection, serum analysis, and 12-month followup. Exclusion criteria: ① Concurrent active cancers, end-stage renal disease, severe liver dysfunction, or hematological disorders; ② Recent use (within 3 months) of drugs altering chemokine expression; ③ Severe mental or cognitive impairment; Pregnant or lactating status.

All patients were treated with the same type of stent and dual antiplatelet therapy. Ethical approval was obtained from the hospital's ethics committee, and written informed consent was acquired from all participants.

### Prognostic follow-up

A minimum one-year prognostic follow-up was completed for all AMI patients, involving monthly visits in the initial six months and visits every two months thereafter. The follow-up period concluded on July 1, 2025, with MACE [12] serving as termination endpoints, including cardiac death, recurrent AMI, target vessel revascularization, or hospitalization due to worsening heart failure.

### Sample collection and detection

Fasting blood (4 mL) was drawn from the ante-cubital vein of both AMI patients (before treatment: before intervention and after treatment: 5 days post-PCI) and control subjects at admission. Samples were collected in coagulation-promoting tubes, allowed to clot at room temperature for 30 minutes, and then centrifuged at 3000 rpm for 15 minutes to isolate serum. The serum aliquots were subsequently stored at -80°C.

Using ELISA kits (Wuhan Cusabio Technology LLC), serum CXCR7 and CXCL12 were measured. Following thawing and vortex mixing, 100 μL of serum samples was aliquoted into the assay wells. The plate layout included blank wells (containing buffer alone), standard wells (with five concentrations in duplicate), and sample wells. After sealing, the plate underwent a 2-hour incubation at 37°C. Subsequently, 100 μL of biotin-conjugated detection antibody was introduced, followed by another 1-hour incubation at 37°C. Color development was initiated by adding 100 μL of streptavidin-HRP solution, and the reaction was terminated prior to measuring the absorbance at 450 nm using a microplate reader. A standard curve was plotted with standard concentrations as the x-axis and absorbance as the y-axis to calculate sample concentrations. Third-party quality controls (Bio-Rad Lyphochek Cardiovascular Disease Control) were used, with low- (LQC, 15 pg/mL) and high-quality control (HQC, 180 pg/mL) controls measured daily before testing, requiring coefficient of variation (CV%) <15%.

NT-proBNP quantification employed an automatic biochemical analyzer (Mindray, BS-2000M). Serum samples (2 μL) were mixed with reagents (200 μL) in reaction cups for automated analysis by the instrument. Daily quality control using Bio-Rad's C-reactive Protein Control Level 1/2 requires running both the LQC (50 pg/mL) and HQC (2000 pg/mL) prior to testing samples, accepting a CV% of less than 5%.

ELISA kits for CXCR7/CXCL12 had intra-assay CV <8% and inter-assay CV <12%. The NT-proBNP assay covered a dynamic range of 10-5000 pg/mL, with samples >5000 pg/mL diluted 1: 5 prior to analysis.

### Statistical analysis

This study used SPSS 30.0 for statistical analysis. Categorical data [n(%)] were compared with the chi-square test. Continuous data are presented as (χ̄±s) and compared with independent or paired t-tests if normally distributed; otherwise, non-normally distributed data are presented as median (P25, P75) and compared using Mann-Whitney U or Kruskal-Wallis H tests. Bonferroni correction was used for multiple comparisons (P<0.017 after correction). Paired t test was used to analyze the dynamic changes of biomarkers. Diagnostic value was analyzed via receiver operating characteristic (ROC) curves. The optimal threshold was determined using the Youden index (Youden index = sensitivity + specificity — 1), balancing maximum sensitivity and specificity, and the AUC along with diagnostic efficiency were ascertained. A logistic regression model was used for joint detection. A statistically significant difference was deemed present at P<0.05

## Results

### Clinical data of AMI patients and controls

No significant differences were observed in age, gender, BMI, etc., between AMI patients and controls (P>0.05, Table 1).

**Table 1:** Baseline characteristics of AMI patients and healthy controls.

Groups	Age	Male vs.<br>Female	Family history<br>of AMI<br>Having vs.<br>not having	Smoking<br>Yes vs. No	Drinking<br>Yes vs. No	Hypertension<br>Having vs.<br>not having	Coronary heart<br>disease<br>Having vs.<br>not having
Control (n = 54)	64.63±6.09	32 vs. 22	6 vs. 48	20 vs. 34	12 vs. 42	30 vs. 24	12 vs. 42
AMI (n = 164)	64.25±7.32	105 vs. 59	22 vs. 138	72 vs. 92	42 vs. 122	102 vs. 62	48 vs. 116
t (or χ^2^)	0.342	0.395	0.730	0.785	0.200	0.750	1.011
P	0.732	0.530	0.393	0.376	0.617	0.387	0.315

### Comparative analysis of CXCR7 and CXCL12

**Figure 1:**
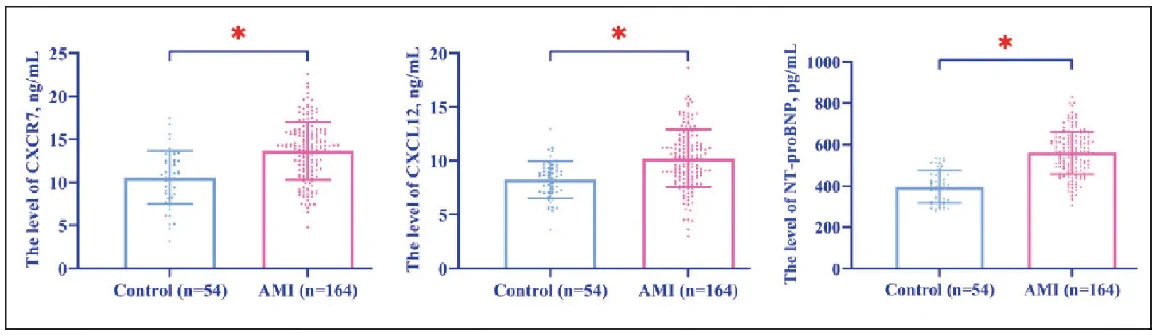
Comparison of the levels of CXCR7, CXCL12 and NT-proBNP * indicates that the difference between the two groups was statistically significant (P < 0.05).

Pre-treatment CXCR7 and CXCL12 in AMI patients were (13.67±3.36) ng/mL,(10.25±2.67) ng/mL, respectively, which were 33.05% and 16.50% higher than those in the control group (P<0.05). Not only that, we noted statistically higher NT-proBNP expression in the AMI group (P<0.05, Figure 1).

### Diagnostic Value of CXCR7, CXCL12, and NT-proBNP for AMI

**Figure 2:**
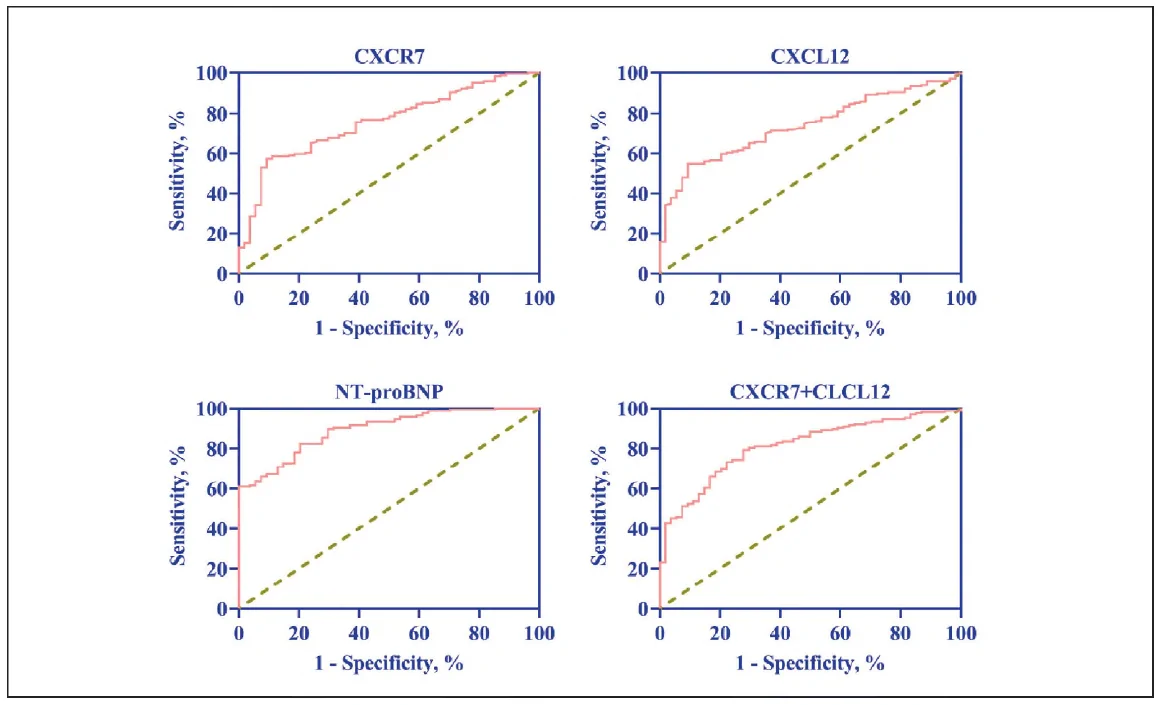
ROC curves of CXCR7, CXCL12, and NT-proBNP for diagnosing AMI.

ROC curve analysis showed that CXCR7, CXCL12, and NT-proBNP each possessed significant diagnostic value for AMI (Figure 2). Combined detection of CXCR7 and CXCL12 further enhanced the diagnostic performance, achieving an AUC of 0.812 with 79.27% sensitivity and 72.22% specificity (P<0.05, Table 2). Although not as excellent as the diagnostic effect of NT-proBNP CXCR7 and CXCL12 still have certain AMI diagnostic effects.

**Table 2:** Effects of CXCR7, CXCL12, and NT-proBNP in diagnosing AMI.

Diagnostic indicators	Cut-off	Sensitivity	Specificity	AUC	95%CI	P
CXCR7	>13.67 ng/mL	57.32	90.74	0.756	0.687-0.825	P<0.001
CXCL12	>10.01 ng/mL	54.88	90.74	0.737	0.669-0.804	P<0.001
NT-proBNP	>457.2 pg/mL	82.32	79.63	0.893	0.850-0.936	P<0.001
CXCR7+CXCL12	>0.6978	79.27	72.22	0.812	0.752-0.873	P<0.001

### Changes in CXCR7, CXCL12, and NT-proBNP before and after AMI treatment

**Figure 3:**
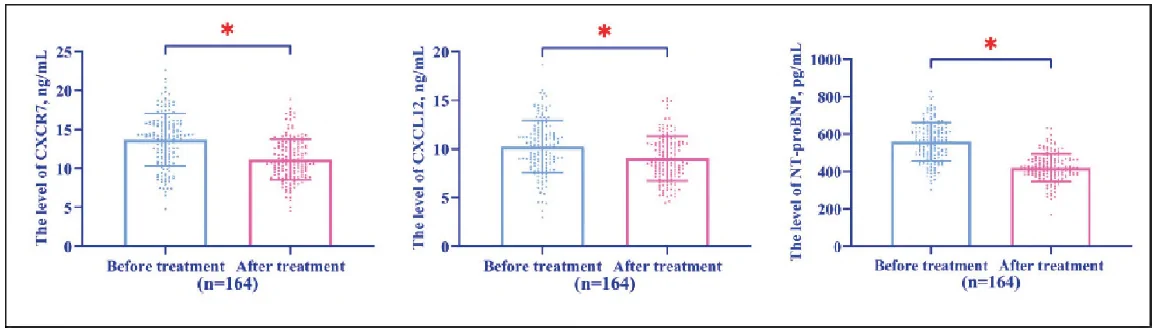
Changes of CXCR7, CXCL12, and NT-proBNP before and after treatment. * indicates that the difference between the two groups was statistically significant (P < 0.05).

Compared with pre-treatment levels, post-treatment CXCR7, CXCL12, and NT-proBNP in AMI patients decreased to (11.13±2.62) ng/mL, (9.04±2.27) ng/mL, and (421.59±73.28) pg/mL, respectively (P<0.05, Figure 3).

### Correlation of CXCR7, CXCL12, and NT-proBNP with MACE

**Figure 4:**
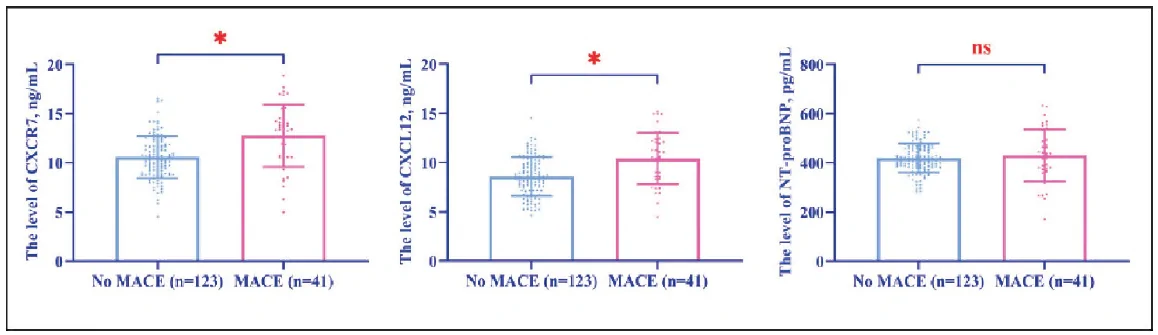
Comparison of CXCR7, CXCL12, and NT-proBNP between MACE patients and non-MACE patients. * indicates that the difference between the two groups was statistically significant (P < 0.05). ns indicated no statistically significant difference between groups (P > 0.05).

All AMI patients were successfully tracked, with a median follow-up duration of [14, (13, 16)] months. MACE was recorded in 30 individuals. Although NT-proBNP levels did not differ significantly between groups (P>0.05), patients who experienced MACE demonstrated markedly higher CXCR7 and CXCL12 concentrations compared to those without such events (P<0.05, Figure 4).

### Diagnostic value of CXCR7 and CXCL12 for MACE

**Figure 5:**
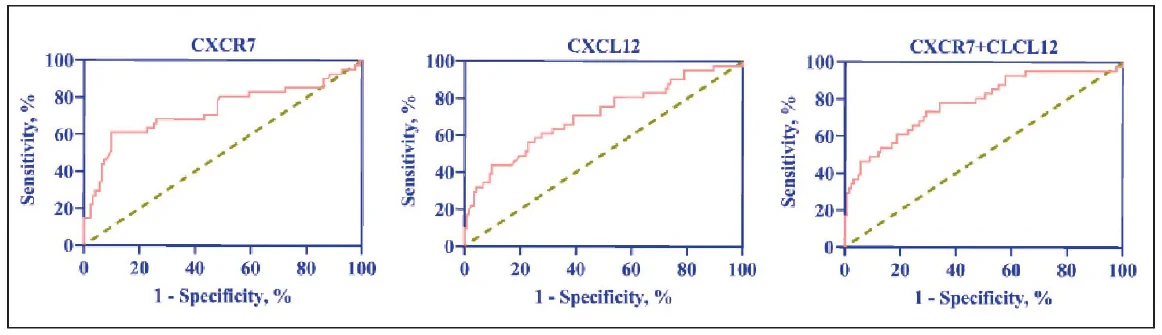
ROC curves of CXCR7 and CXCL12 for diagnosing MACE.

ROC curve analysis of post-treatment CXCR7 and CXCL12 levels showed good diagnostic efficacy of both markers for predicting MACE in post-PCI AMI patients (Figure 5). When combined, 78.05% sensitivity and 65.85% specificity were achieved for MACE prediction (AUC = 0.779), suggesting high clinical reference value (Table 3).

**Table 3:** The effects of CXCR7 and CXCL12 in diagnosing MACE.

Diagnostic indicators	Cut-off	Sensitivity	Specificity	AUC	95%CI	P
CXCR7	>13.10 ng/mL	60.98	90.24	0.729	0.625-0.833	P<0.001
CXCL12	>11.05 ng/mL	43.90	90.24	0.707	0.609-0.805	P<0.001
CXCR7+CXCL12	>0.2206	78.05	65.85	0.779	0.691-0.867	P<0.001

## Discussion

As a leading cause of death from cardiovascular diseases globally, AMI has seen a rising incidence in recent years [13]. MACE, which significantly impact patient prognosis after AMI, also continue to pose a substantial risk [14]. Herein, we investigate the diagnostic potential of CXCR7 and CXCL12 for AMI and MACE, proposing a new strategy for clinical assessment.

Initial analysis revealed elevated pre-treatment CXCR7 and CXCL12 levels in AMI patients compared to healthy controls, aligning with previous basic research [15][16]. These findings imply a pivotal involvement of the CXCR7/CXCL12 axis in AMI development. Moreover, the combined use of both biomarkers demonstrated superior diagnostic performance (AUC = 0.812) over single-marker measurements, suggesting a potential synergistic relationship between CXCR7 and CXCL12. Hence, the dual regulatory role of the CXCR7/CXCL12 axis in AMI warrants in-depth exploration. Basic studies show that CXCL12 binding to its receptor CXCR7 triggers PI3K/Akt pathway activation. This stimulation enhances endothelial progenitor cell (EPC) proliferation and migration, fostering angiogenesis in the peri- infarct area [17]. Furthermore, this ligand-receptor binding exerts an inhibitory effect on the NF-kB pathway, leading to diminished neutrophil recruitment and the attenuation of a pro-inflammatory cytokine cascade, including factors like IL-6 and TNF-α [18]. Additionally, decreased CXCR7 and CXCL12 after PCI in AMI patients suggests that PCI may reduce localized passive CXCL12 release by mechanically restoring vessel patency, while active CXCR7 down-regulation marks the commencement of myocardial tissue repair. This is consistent with the study of Ghadge et al. [19] Ghadge et al [19] found that CXCR7 affected cardiac capillary density reduction and fibrosis area increase in mice with coronary ischemia and cardiac hypertrophy.

Furthermore, concentrations of both CXCR7 and CXCL12 were higher in patients who experienced post-PCI MACE compared to those who did not. CXCR7 plus CXCL12 measurement showed good diagnostic value for predicting MACE, identifying them as independent predictors of post-PCI MACE. This finding echoes prior animal experiments, where CXCR7-knockout mice were more prone to ventricular remodeling due to impaired myocardial repair [10]. Specifically, sustained high CXCL12 expression may promote adverse outcomes through two pathways: ① CXCR7 overactivation triggers endothelial-mesenchymal transition (EndMT) in EPCs, impairing their ability to form new blood vessels; ② Elevated post-PCI CXCR7/CXCL12 may paradoxically promote in-stent restenosis through CXCR7-mediated smooth muscle cell proliferation [20]. This aligns with our observation of higher MACE rates in patients with persistent biomarker elevation.

NT-proBNP a traditional myocardial injury marker, showed significant diagnostic efficacy for AMI but no correlation with MACE in the present study. This discrepancy may be attributed to two reasons. First, NT-proBNP primarily reflects acute myocardial injury severity, whereas MACE is more closely related to chronic inflammation, impaired vascular repair, and endothelial dysfunction [21]. Second, NT-proBNP secretion is affected by various confounding factors such as renal function and volume load, particularly over extended follow-up periods [22]. In comparison, CXCR7/CXCL12 directly participate in dynamic regulation of the coronary microenvironment and better capture the pathophysiology of MACE, leading to superior specificity and sensitivity in MACE prediction.

Based on the study's findings, incorporating CXCR7 and CXCL12 into the risk assessment system for post-PCI patients is recommended. Clinical translation should consider serial measurements at postPCI days 5 (baseline), 30, and 90 to track microenvironment remodeling dynamics. Clinically, tracking the dynamic changes in these biomarker levels allows for early assessment of post-prognostic MACE risk. In patients identified as high-risk based on persistently elevated CXCR7/CXCL12, intensified anti-inflammatory therapy (e.g., colchicine) or targeted vascular repair therapy may be particularly advantageous. However, several limitations are noted: (1) the single-center design (n = 162 patients from a single hospital), which may limit generalizability. Regional variations in AMI management (e.g., reperfusion times, adjunctive therapies) and genetic backgrounds may influence biomarker performance. Multi-center validation is urgently needed; (2) short-term follow-up (median 14 months), insufficient to assess long-term MACE risk; and (3) lack of validation in external cohorts. Multi-center studies with larger samples and prolonged follow-up are needed.

## Conclusion

The combined detection of CXCR7 and CXCL12 exhibits a favorable prognostic utility in diagnosing AMI and predicting post-PCI MACE. Unlike conventional biomarkers, CXCR7/CXCL12 more accurately mirrors the dynamic repair processes within the coronary microenvironment, offering novel insights for personalized risk assessment.

## Dodatak

### Consent to publish

All authors gave final approval of the version to be published.

### Availability of data and materials

The data that support the findings of this study are available from the corresponding author upon reasonable request.

### Funding

This study was supported by the Shanghai Pudong New Area Health System Outstanding Young Medical Talents Training Program(No.PWRq2023-08) and Shanghai Pudong New Area Important Weak Discipline Construction Project(No.PWZbr2025-09).

### Acknowledgements

Not applicable.

### Author contributions

JW.S. conceived and designed the project, H.P and XL.F wrote and revised the paper, Y.L generated and analyzed the data, G.A. supervised the study, H.P and XL.F. made equal contributions in this work as co-first authors.All authors gave final approval of the version to be published, and agree to be accountable for all aspects of the work.

Hong Pan and Xiaoli Fan contributed equally to this work.

### Conflict of interest statement

All the authors declare that they have no conflict of interest in this work.
